# *In Vitro* Inhibition of Hepatitis C Virus by Antisense Oligonucleotides in PBMC Compared to Hepatoma Cells

**DOI:** 10.1155/2014/196712

**Published:** 2014-06-01

**Authors:** Samar Samir Youssef, Ahmed Mohamed Fahmy, Moataza Hassan Omran, Amr Saad Mohamed, Mohamed Ali El Desouki, Mostafa K. El-Awady

**Affiliations:** ^1^Microbial Biotechnology Department, National Research Center, Cairo 12311, Egypt; ^2^Reproductive Health and Family Planning Department, National Research Center, Cairo, Egypt; ^3^INRS-Institut Armand Frappier, Laval, QC, Canada H7V 1B7; ^4^Chemistry Department, Faculty of Science, Cairo University, Cairo 12613, Egypt

## Abstract

*Aim.* To assess the efficiency of phosphorothioate antisense oligodeoxynucleotide 1 (S-ODN1) on HCV translation inhibition in PBMC compared to hepatoma cells *in vitro* for the first time. *Materials and Methods.* The study included 34 treatment naive HCV patients. IRES domain III and IV sequence variations were tested in 45 clones from 9 HCV patients. PBMC of HCV positive patients were subjected to S-ODN *in vitro*. Concomitantly HepG2 cells infected by the same patient's serum were also treated with S-ODN1 for 24 and 48 hours. Cellular RNA was tested for HCV plus and minus strands by reverse transcription polymerase chain reaction (RT-PCR). *Results.* Sequence variations were seen in HCV IRES domain III only while domain IV was conserved among all the tested patient's clones. S-ODN1 successfully inhibited HCV translation in HepG2 cells, while in PBMC inhibition was partial. *Conclusion.* HCV IRES domain IV is more conserved than domain IIId in genotype 4 HCV patients. S-ODN against HCV IRES domain IV was not efficient to inhibit HCV translation in PBMC under the study conditions. Further studies testing other S-ODN targeting other HCV IRES domains in PBMC should be done.

## 1. Introduction

Chronic hepatitis C virus (HCV) infection is a leading cause of chronic hepatitis, liver cirrhosis, and hepatocellular carcinoma [1–3]. The World Health Organization estimated that 3% of the world's population or approximately 130–170 million people were chronically infected with HCV at the end of the 20th century, and 2.3–4.7 million new infections occur per year. Hepatitis C virus is also responsible for 300 000 deaths annually [4]. HCV mainly is a hepatotropic virus with proven lymphotropism [5–7]. These cells represent an extrahepatic reservoir that can be implicated in virus recurrence and persistence [8, 9]. Clearance of HCV RNA in peripheral blood mononuclear cell is a predictor of response to antiviral therapy in patients with chronic hepatitis C [10].

Six major genotypes of HCV have been identified worldwide [11]; HCV genotype 4 (G4) is common in the Middle East and Egypt [12, 13] and has become increasingly prevalent in Southern European countries [14, 15]. Current standard therapy for G4 HCV infected patients is a 48-week course of pegylated interferon (Peg-IFN) and ribavirin (RBV). The treatment goal is a sustained virologic response (SVR), defined as an undetectable HCV RNA load 6 months after treatment cessation. The SVR rate for G4 HCV infected patients after Peg-IFN/RBV treatment is approximately 60% [16]. Currently, novel compounds against the HCV-NS3 protease or the HCV-NS5B RNA-polymerase have entered clinical trials, showing high antiviral potency [17]. However, rapid HCV drug resistance to these agents has been shown to limit their efficacy, necessitating a combination with PEG-IFN and ribavirin, which may cause a wide range of serious adverse reactions [18, 19]. Consequently, antisense technologies are still needed in the future therapy for HCV. The 5′-end of the HCV genome contains a noncoding region (5′NCR) of 341 nt, which is the most highly conserved region among all HCV strains [20]. It forms a stable secondary structure which contains an internal ribosome entry site (IRES) necessary for HCV translation/replication, representing an ideal target for antisense approaches.

Phosphorothioate modified ODN (S-ODN) is the first generation of antisense drugs entering clinical trials for the treatment of patients with chronic HCV infection which show acceptable properties for drug development [21].

Previous studies from our lab and others have shown the successful* in vitro* inhibition of HCV translation by antisense oligonucleotides directed towards the IRES [17, 22–26]. In our previous study and that of Alt et al. [22, 23], higher efficiency of S-ODN1 (directed towards IRES IId domain) compared to S-ODN2 (directed towards domain IRES IV domain) was reported but was not justified. In addition, S-ODN effect on HCV translation in HCV positive peripheral blood mononuclear cells (PBMCs) has not been investigated so far. The aim of this study was to evaluate the* in vitro* efficiency of S-ODN1 to inhibit HCV translation in patient's PBMC compared to hepatoma cells (HepG2), moreover, and to investigate sequence based reasons for higher efficiency of S-ODN1 versus S-ODN2.

## 2. Materials and Methods

### 2.1. Patients and Samples Processing

A total of 34 patients were included in the study; they were naïve newly diagnosed HCV patients from the Medical Unit at the National Research Center, Cairo, Egypt. All patients were HCV antibody positive by third generation ELISA. Sera from 9 HCV infected subjects were used in structural analysis of IRES domain III (loop IIId), while the rest twenty-five subjects were subjected to HCV detection in serum and PBMC in order to be further used for comparing S-ODN1 inhibition efficiency in HepG2 versus PBMC* in vitro*. The study was approved by the ethical committee of the National Research Center and a signed consent form was obtained from each patient. Patient's inclusion criteria were to be monoinfected by HCV detected by HCV antibodies (Ab) in serum and not suffering other disorders and not diabetic.

Ten mL blood was withdrawn from each patient, 7 mL on heparin and 3 mL without anticoagulant from which serum was collected by centrifugation. Ficoll centrifugation was used to isolate plasma and PBMC from blood. Plasma was collected and stored as aliquots in three Eppendorf tubes; PBMC was collected, washed with PBS five times, resuspended in RPMI 1640 media, and counted; then part of it (2 × 10^6^ cells) was used for detection of HCV and the rest was plated in 6 well plate (1 × 10^6^ cells/well) for testing SODN.

### 2.2. S-ODN Choice and Synthesis

According to previous reports including one from our lab [22, 23], efficient inhibition of viral replication* in vitro* was accomplished by two S-ODN, namely, S-ODN1 (nt 326–348) and S-ODN2 (nt 254–272), targeted towards HCV IRES domain IIId and IV, respectively. S-ODN1^∗^ has the same sequence of S-ODN1 with one mismatched nucleotide introduced (underlined nucleotide);

 S-ODN1: (5′TGCTCATGGTGCACGGTCTACGA3′)
 S-ODN1^∗^: (5′TGCTCTTGGTGCACGGTCTACGA3′)
 S-ODN2: (5′GGCCTTTCGCGACCCAA3′).

Antisense phosphorothioate nucleotides were prepared and highly purified by Biognostik, Gesellschaft fur molekulare diagnostik, Gottingen, Germany.

### 2.3. Detection of HCV in Patient's Sera

Two-hundred-microliter (*μ*L) serum from each patient was used for RNA extraction using QIAamp Viral RNA kit from QIAGEN (USA) according to the manufacture's manual. Two hundred ng of RNA was used to detect HCV by one step RT-PCR kit purchased from QIAGEN (USA) and used according to the manufacture's protocol. PCR products were visualized on 2% agarose gel.

### 2.4. HCV Genotyping in Serum and PBMC

Genotyping of HCV was done using nested PCR amplification of HCV core gene with genotype specific primers according to Ohno et al. [27]. Amplified PCR products were visualized on 2% agarose gel.

### 2.5. Analysis of IRES Domain III Sequence from Local Samples

HCV domain III sequence was amplified by RT-PCR, then purified, and cloned as described in our previous study [28]. The ClustalW program was used (http://www.ch.embnet.org/software/ClustalW.html) for alignment of the obtained sequences with genotype 4 prototype.

### 2.6. Analysis of IRES Domain IV Sequence from Local Samples

Published sequences of Egyptian HCV genotype 4 subjects with accession numbers AY838804, AY838805, AY838806, AY838807, Y838808, Y838809, AY838810, AY838811, AY838812, and AY838813 obtained from NCBI gene bank (http://www.ncbi.nlm.nih.gov/) and available at HCV database (http://www.hcv.lanl.gov/) were aligned and analyzed using ClastalW and biological sequence editor BioEdit programs for multiple sequence alignment and results were confirmed by the entropy plot generated by BioEdit program.

### 2.7. Detection of HCV Strands in Patient's PBMC

Total cellular RNA was extracted from counted uncultured patient's PBMC using Biozol reagent (Bioer, China) according to the manufacture's manual. HCV strands were detected by RT-PCR as previously described [29]; briefly, 200 ng of RNA was reverse transcribed to cDNA in 25 *μ*L reaction mixture containing 20 U of AMV reverse transcriptase (Promega, Madison, WI, USA), 200 *μ*M of each dNTP, and 25 pmoles of either antisense primer (1CH: 5′-GGT GCA CGG TCT ACG AGA CCT-3′) for plus strand or sense primer (2CH: 5′-AAC TAC TGT CTT CAC GCA GAA-3′) for minus strand. The first round PCR was done in a total volume of 50 *μ*L using 50 pmoles from each primer (2CH and P2: 5′-TGC TCA TGG TGC ACG GTC TA-3′). Twenty percent of the reaction was used for a second amplification round with the internal pair of primers (D1: 5′-CGC AGA AAG CGT CTA GCC AT-3′ and D2: 5′-ACT CGG CTA GCA GTC TCG CG-3′). Cycling conditions on the thermal cycler were the same as the first round. Products of PCR were analyzed on 2% agarose gel electrophoresis and photographed.

### 2.8. Testing of S-ODN Inhibitory Effect in Different Cell Types

Patients proved to be HCV positive in both serum and PBMC were included in this step. For each patient, 500 *μ*L serum was used for infected cultured HepG2 cells as previously described [22]; then cells were treated with 1 *μ*M SODN1 and SODN1^∗^ (4 wells for each treatment) for 72 hr; control cells were kept without SODN. Concomitantly, PBMCs were separated by ficoll, washed 5 times with PBS, then resuspended in RPMI 1640 supplemented with 10% FCS, plated on a 6-well plate at a concentration of 2 × 10^6^/mL, and cultured at 37°C, 5% CO2 in absence or presence of 1 *μ*M SODN1 and SODN1^∗^ (4 wells for each treatment) for 72 hr. Cells were harvested, washed 5 times with PBS, and subjected to total cellular RNA extraction. Total RNA extracted from cultured HepG2 cells and from PBMC was reverse transcribed and amplified using same assay described above and previously described to detect HCV plus and minus strand [29].

## 3. Results

### 3.1. HCV Genotyping in Serum and PBMC

Results of HCV genotyping in sera of all patients showed that all were genotype 4. Similarly, genotype 4 was the only genotype detected from genotyping of HCV from PBMC positive patients included.

### 3.2. HCV IRES Domain III and IV Sequence Conservation

**Figure 1:**
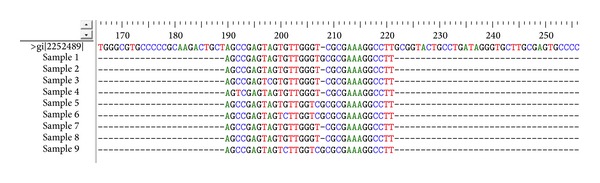
Multiple sequence alignment of AUG domain using ClustalW program. After sequencing, nine sequences comprising the AUG (34 nucleotides) were aligned using ClastalW program. The figure represents conservation of aligned sequences.

**Figure 2:**
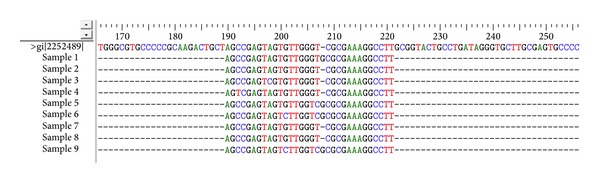
Multiple sequence alignment of loop IIId using ClustalW program. After sequencing, nine sequences comprising loop IIId were aligned using ClustalW program. Figure represents lower conservation with mutations C3-T3 in sample 4, A9-C9 for sample 3, G12-C12 for samples 6 and 9, G17-T17 for samples 5, 6, and 9, T18-C18 for samples 5, 6, and 9, and G19 insertion for samples 1, 5, 6, and 9.

To assess IRES sequence conservation across HCV isolates, nucleotide sequences of 45 clones from 9 HCV subjects were determined (and the most frequent sequence for the clones that belongs to each subject was selected) and the sequences that constitute the target domains for S-ODN1 and S-ODN2 were aligned using multiple sequence alignment programs ClustalW. Results revealed sequence conservation (Figure 1) at the region containing the AUG start codon (34 nucleotides), which was selected as target for S-ODN1, while stem loop IIId sequence, which was selected as a target for S-ODN2, was found to be relatively variable through multiple sequence alignment by ClustalW program (Figure 2).

### 3.3. Detection of HCV RNA in Patient's Serum and PBMC

All the 25 patients were HCV RNA positive in serum but only 10 out of them showed detectable HCV RNA in PBMC. Among them six patients showed detectable plus and minus HCV RNA strands while the rest 4 patients showed the presence of HCV plus strand only.

### 3.4. *In Vitro* Inhibition of HCV Replication Using S-ODN1 in Both Infected HepG2 Cell Line and PBMC

**Figure 3:**
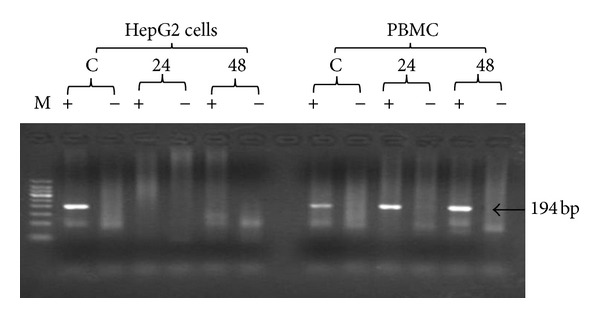
Agarose gel electrophoresis HCV nested PCR products in two different types of cells treated with S-ODN1. HCV infected HepG2 cells and PBMC were treated with SODN1 for 24 and 48 hours. Lane M: 50 bp DNA ladder. For HepG2 cells, lanes C (+, −): untreated HCV infected HepG2 cells cultured for 48 h amplified plus and minus strands, respectively, as a positive control, lanes 24 h (+, −): plus and minus strands PCR products, respectively, for HCV infected HepG2 cells treated with S-ODN1 for 24 h, and lanes 48 h (+, −): plus and minus strands PCR products, respectively, for HCV infected HepG2 cells treated with S-ODN1 for 48 h. For PBMC, lanes C (+, −): plus and minus strands PCR products, respectively, for untreated HCV infected PBMC cultured for 48 h as a positive control, lanes 24 h (+, −): plus strand and minus strands PCR products, respectively, for HCV infected PBMC treated with SODN1 for 24 h, and lanes 48 h (+, −): plus strand and minus strands PCR products, respectively, for HCV infected PBMC treated with S-ODN1 for 48 h.

In the current study inhibition of HCV was defined as elimination of preexisting viral strand/s. Results showed that supplementation of the culture with S-ODN1 succeeds to inhibit HCV RNA strand/s in serum infected HepG2 cells after 24 hours at 1 *μ*M concentration and its effect was extended till 48 hours in all tested cases except one, while in PBMC S-ODN1 was considered to fail in inhibition of preexisting HCV RNA strand/s (Figure 3) after 24 and even after 48 hours in all patients tested, although partial inhibition of HCV manifested by loss of HCV minus strand in 2 patients after 48 hr and in one patient after 24 hr (Table 1) and loss of HCV plus strand in one patient after 24 hr (Table 1). Complete viral inhibition was not detected in any of them.

### 3.5. Effect of Variation of S-ODN1 Sequence on HCV Inhibition in Different Cell Types

**Figure 4:**
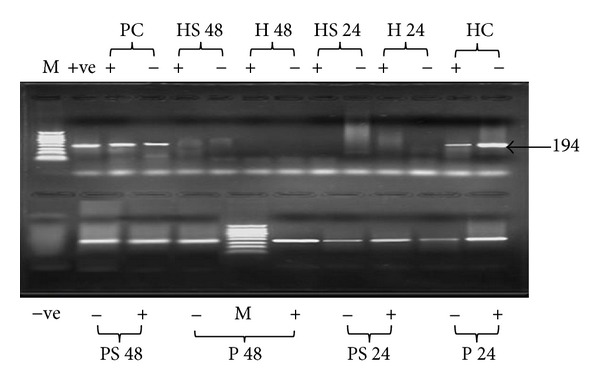
Agarose gel electrophoresis of HCV nested PCR products in two different types of cells treated with S-ODN1 and its derivative. HCV infected HepG2 cells and PBMC were treated with S-ODN1 for 24 and 48 hours. Lanes HC (+, −): untreated HCV infected HepG2 cells cultured for 48 h amplified plus and minus strands, respectively, and lanes H 24 (+, −): plus and minus strands PCR products, respectively, for HCV infected HepG2 cells treated with S-ODN1 for 24 h, lanes HS 24 (+, −): plus and minus strands PCR products, respectively, for HCV infected HepG2 cells treated with S-ODN1 derivative for 24 h, lanes H 48 (+,−): plus and minus strands PCR products, respectively, for HCV infected HepG2 cells treated with S-ODN1 for 48 h, lanes HS 48 (+, −): plus and minus strands PCR products, respectively, for HCV infected HepG2 cells treated with S-ODN1 derivative for 48 h, lanes PC (+, −): untreated HCV infected PBMC cultured for 48 h amplified plus and minus strands, respectively,* +ve*: positive PCR control, and lanes P 24 (+, −): plus and minus strands PCR products, respectively, for HCV infected PBMC treated with S-ODN1 for 24 h, lanes PS 24 (+, −): plus and minus strands PCR products, respectively, for HCV infected PBMC treated with S-ODN1 derivative for 24 h, lanes P 48 (+, −): plus and minus strands PCR products, respectively, for HCV infected PBMC treated with S-ODN1 for 48 h, lanes PS 48 (+, −): plus and minus strands PCR products, respectively, for HCV infected PBMC treated with S-ODN1 derivative for 48 h,* –ve*: is negative PCR control.

After testing S-ODN1 efficiency in different cell types. Effect of S-ODN1 sequence conservation on its inhibitory effect of HCV replication results is shown in Figure 4; one nucleotide mismatch in S-ODN 1 did not show effect on inhibition efficiency of the antisense oligonucleotide; furthermore, both S-ODN1 and its one nucleotide mismatched derivative were able to inhibit replication in HepG2 cells while both failed to inhibit replication in PBMC.

## 4. Discussion

Several groups including our laboratory have identified antisense oligonucleotides that inhibit HCV RNA and polyprotein synthesis both* in vitro*, cell culture, and in mouse models [17, 22, 23, 26, 30–34].

Our laboratory previously used S-ODN structures designed against two phylogenetically conserved regions, the region comprising the AUG start codon (S-ODN1) and stem loop IIId (S-ODN2) [22]. The sequence data from local isolates revealed conservation at specific motifs related to proper folding and efficient translation, that is, IIId GGG (nucleotide 266–268) and AUG start codon (nucleotide 340–342), respectively. These data offered an advantage for antisense drugs as a therapeutic option for most known genotypes of HCV. One important and unjustified finding in this study is that S-ODN1 has relatively more inhibitory potency than S-ODN2, a finding that was supported earlier [23]. On the other hand, ISIS pharmaceuticals achieved a progression in transfer of the* in vitro* results to* in vivo* trials studies in a phase I clinical trial of ISIS14803 product in patients with chronic hepatitis C infection who had previously not responded to therapy or were unsuitable candidates for standard therapies. ISIS study was designed to evaluate the safety and tolerability of four ISIS 14803 doses given by two routes of administration although the study was not focused on detecting significant changes in antiviral response rates between cohorts. In general ISIS safety study was accompanied by ALT flares in a subgroup of patients. Transient plasma HCV RNA reductions greater than fluctuations generally seen in untreated patients were observed in 3 of 28 ISIS 14803-treated patients [21].

In the present study IRES domain III specifically the segment starting from nucleotide 141 to 279 derived from 9 different naïve randomly selected Egyptian patients exclusively infected with genotype 4 was amplified. Cloning of domain III and sequencing of 5 clones in each patient allowed the identification of the major variant (identical sequence in 4 clones or more) in each patient so that noise was reduced during extrapolation of the relationship between genomic variation and efficient selection of the most specific antisense nucleotide sequence.

Alignment of most frequent sequences belonging to each of the nine patients have shown that domain IV is highly conserved among all nine selected sequences specially the 34-nucleotide stretch that comprises the AUG start codon; on the other hand loop III d showed a lower sequence conservation which may give a strong evidence on the higher efficiency of S-ODN1 which is directed against domain IV over S-ODN2 which is directed against loop III d to arrest viral replication. Nonetheless, in our earlier study [22] alignment of 5′ UTR sequences was done for 17 clones from 5 genotype 4 patients only, and results showed nucleotide differences ranging from 3.5% to 5.3% when compared to isolates of type 4 used in this study; the two stem loop targets for S-ODN1 and S-ODN2 were conserved among all isolates analyzed except for a single mismatch in only one isolate.

The presence of HCV in extrahepatic sites may have profound implications in the pathogenesis of hepatitis C and treatment outcome. Evidence of HCV replication in granulocytes, monocytes, macrophages, dendritic cells, and B lymphocytes has been reported, although data were inconsistent. Further evidence for extrahepatic replication of HCV was provided by modeling of viral kinetics in patients during liver transplantation [6, 35].

Many studies have provided evidence on the existence of HCV replication in PBMC by the detection of HCV RNA minus strand the putative replication intermediate using strand-specific RT-PCR. Furthermore, PBMC was also considered a reservoir for HCV particles. Supporting evidence was given as fast viral decay in PBMC was associated with SVR [36]. Another study has shown the direct relation between SVR and absence of HCV RNA minus strand in PBMC [37].

In the current study naïve randomly selected local Egyptian patients exclusively infected with genotype 4 from which PBMCs were taken were selected and also the same patient serum was used to infect HepG2 cells in order to exclude viral heterogeneity effect and to narrow the focus on the efficiency of the treatment with S-ODN1 assuming that the serum and PBMC of the same patient will have the same variants.

Effect of mismatched oligonucleotides on HCV RNA levels was also studied by using S-ODN1 altered sequence after random introduction of a single nucleotide substitution which subsequently showed no effect on the potency of S-ODN1 to inhibit HCV replication, therefore, suggesting that S-ODN1 retains its high efficiency even after single base substitution.

Results of this study have shown that S-ODN1 was efficiently able to inhibit viral replication in infected HepG2 cells as indicated by the absence of plus and/or minus strands while, in contrast, it failed to inhibit viral replication in PBMC. These results suggested few explanations for the apparent cell tropism. The first explanation is the existence of distinct HCV variants in PBMC different from these present in hepatoma cells, a speculation based on results from previous studies which proved that tissue-specific mutations often occur in the HCV IRES, presumably because the cellular proteins that facilitate IRES function differ between cell types, driving the evolution of variants adapted for the local set of proteins [38]. The second explanation is that the concentration of S-ODN1 used was not sufficient to stop HCV translation in PBMC due to insufficient uptake of SODN by PBMC as no delivery system was used to enhance uptake of SODN by PBMC, consequently further studies in which a delivery system is used are recommended; while the third explanation may be that the translation initiation proteins in PBMC which physically interact in the translational complex for access to target may cause distinct secondary and tertiary structure changes in the RNA which differ from those in hepatoma cells and can prevent stringent oligonucleotide hybridization. However, this is one of several other viral and host factors that will determine translational efficiency and possibly resistance to treatment that need to be studied extensively in further studies.

In conclusion, S-ODN1 is efficient to inhibit HCV translation in hepatoma cells only and not in PBMC as extrahepatic translation site; consequently the use of antisense as antiviral treatment for HCV might necessitate its combination with PEGIFN/RBV in patients proved to harbor extrahepatic viral existence. The deficiency in the current study is the lack of data regarding the sequence of HCV in the studied patients. However, results of the current study urges the need for further studies on other HCV genotypes and with larger samples number to confirm results.

## Figures and Tables

**Table 1:** *In  vitro* effect of SODN1 on HCV RNA in PBMC.

	Control		PBMC			
Patient number			24 hr		48 hr	
	+ve strand	−ve strand	+ve strand	−ve strand	+ve strand	−ve strand
1	+ve	−ve	+ve	−ve	+ve	−ve
2	+ve	*+ve *	+ve	+ve	+ve	**−ve**
3	+ve	−ve	+ve	−ve	+ve	−ve
4	+ve	+ve	+ve	+ve	+ve	+ve
5	+ve	*+ve *	+ve	**−ve**	+ve	+ve
6	*+ve *	+ve	**−ve**	+ve	+ve	+ve
7	+ve	+ve	+ve	+ve	+ve	+ve
8	+ve	−ve	+ve	−ve	+ve	−ve
9	+ve	+ve	+ve	+ve	+ve	+ve
10	+ve	*+ve *	+ve	+ve	+ve	**−ve**
